# Associations Between Quality of Life, Psychosocial Well-being and Health-Related Behaviors Among Adolescents in Chinese, Japanese, Taiwanese, Thai and the Filipino Populations: A Cross-Sectional Survey

**DOI:** 10.3390/ijerph17072402

**Published:** 2020-04-01

**Authors:** Regina L.T. Lee, Wai Tong Chien, Jason Ligot, Jennifer M. Nailes, Keiko Tanida, Sachi Takeuchi, Masanori Ikeda, Sachiyo Miyagawa, Toshisaburo Nagai, Rutja Phuphaibul, Chatsiri Mekviwattanawong, Ying-Ya Su, Rui Xing Zhang, Paul H. Lee, Stephen W. H. Kwok

**Affiliations:** 1 School of Nursing and Midwifery, Faculty of Health and Medicine, University of Newcastle, Callaghan, New South Wales 2308, Australia; 2 The Nethersole School of Nursing, Faculty of Medicine, The Chinese University of Hong Kong, Hong Kong, China; wtchien@cuhk.edu.hk; 3 College of Public Health, University of the Philippines Manila, Manila 1000, Philippines; jdligot@up.edu.ph; 4 Research Institute for Health Sciences, University of the East Ramon Magsaysay, Memorial Medical Center, Quezon City 1113, Philippines; jmnailes@uerm.edu.ph; 5 College of Nursing Art & Science, University of Hyogo, 13-71, Kitaojicho, Akashi, Hyogo 673-8588, Japan; keiko_tanida@cnas.u-hyogo.ac.jp (K.T.); sachi_takeuchi@cnas.u-hyogo.ac.jp (S.T.); masanori_ikeda@cnas.u-hyogo.ac.jp (M.I.); 6 Faculty of Education, Osaka Medical College, Takatsuki City, Osaka 569-8686, Japan; 7 Faculty of Education, St. Andrew’s University of Education, Osaka 590-0114, Japan; t-nagai@andrew-edu.ac.jp; 8 Ramathibodi School of Nursing, Faculty of Medicine, World Health Organization Collaborating Center, Mahidol University, Bangkok 10400, Thailand; ruja.phu@mahidol.ac.th (R.P.); chatsiri.mek@mahidol.edu (C.M.); 9 Taiwan School Nurses’ Association, 800 Kaohsiung, Taiwan; snac819@ms76.hinet.net; 10 School of Nursing, Zhengzhou University, 100 Kexue Dadao, Zhengzhou 450001, China; zhangruixing@zzu.edu.cn; 11 School of Nursing, World Health Organization Collaborating Center for Community Health Services, The Hong Kong Polytechnic University, Hong Kong, China; paul.h.lee@polyu.edu.hk (P.H.L.); wai-hang-stephen.kwok@connect.polyu.hk (S.W.H.K.)

**Keywords:** adolescents, socio-demographic factors, lifestyle, domains of psychosocial well-being, mental health, quality of life, Asia Pacific region

## Abstract

Health-related behaviors during adolescence have lifelong impacts. However, there are unclear areas regarding the associations between health-related quality of life and demographic characteristics, as well as physical and psychosocial indicators. The aim of this study was to examine the associations between quality of life and body weight, sleep outcome, social support by age, and cohabitants, given that income, self-esteem, lifestyle, emotional, social and behavioral problems were taken into account among adolescents in East and Southeast Asia. A cross-sectional survey was conducted in Zhengzhou of China, Hong Kong, Kansai region of Japan, Taipei of Taiwan, Bangkok of Thailand and Manila of the Philippines between 2016 and 2017 among 21,359 urban adolescents aged between 9 and 16. The results showed that adolescents who had better self-esteem and control of emotions and behaviors had much higher level of perceived quality of life. Those who were overweight or obese, sleepy in the daytime, and not living with parents had worse quality of life compared with those who were not. In conclusion, psychosocial well-being should have a higher priority in the promotion of quality of life among Asian adolescents. Nevertheless, further studies are required to explore the differences in perceived quality of life between genders and countries.

## 1. Introduction

The World Health Organization [1] defines adolescence as the period of life between 10 to 19 years. Around one fifth of the world’s population are adolescents. Childhood and adolescence are marked by an overall physical growth spurt [2]. Adolescents’ unique developmental needs during puberty put them on an array of specific health concerns. Adolescence is a critical period in human health and development. At the same time, adolescence provides an opportunity to address health issues that arose during childhood, making it a critical period for health protection and promotion.

Health-related behaviors of adolescents have been a concern in public health for the past two decades in the Western Pacific Region. According to the latest results from the Global Burden of Diseases 2016 Risk Factors Collaborators [3], several modifiable risk factors, such as alcohol use, unsafe sex, and high body mass index contributed to 12.8, 1.02 and 0.15 years of life lost (YLLs) per 100,000 population, respectively, among adolescents aged between ten to fourteen in high-income Asia Pacific regions in 2016. Adverse events, exposure to violence and injuries, health-related behavior, and the overall state of health during adolescence will have physical and psychosocial impacts on the rest of the life course.

Mental health is particularly important during adolescence as many symptoms of mental illness begin to manifest during this period and may go unrecognized. The World Health Organization [4] proposed fifty Sustainable Development Goals (SDG) that are indicators of human development. Under the SDG Goal 3 (good health and well-being), one of the targets is to reduce premature mortality from non-communicable diseases (NCDs) by promoting mental health and well-being. In addition, promoting adolescent health is one of the WHO’s priorities in the period of 2018–2022 and this was announced at the WHO Assembly Meeting in Geneva. The health-related SDG indicators have been grouped into seven thematic areas including risk factors of NCDs and mental health to address the country-specific health issues. Thus, it is important to address NCDs and their risk factors and make recommendations to promote adolescent health in the global action framework.

Quality of life is one of the important constructs that reflect the health and well-being of adolescents. The domains of quality of life, psychosocial, and mental health indicators were found to be intercorrelated in the literature [5,6,7,8,9,10,11,12,13], and these indicators at adolescence were associated with their well-being in adulthood [8,14,15]. In previous studies, the demographic characteristics such as family-related factors have been shown to be associated with quality of life and psychosocial health conditions among adolescents [16,17,18]. Lee, et al. [19] found that adolescent lifestyle and social support were positively associated with quality of life and were different between Asian-Pacific countries. Nowadays, social and cultural changes have created challenges for adolescents to assume roles and responsibilities in health promotion.

In the greater Chinese population, the Han ethnicity is the largest ethnic group (more than 90%) who reside in China, Taiwan and Hong Kong. In China, Xin, et al. [20] conducted a cross-temporal meta-analysis of 56 studies and found that the decreasing trend of Chinese adolescents’ mental health issues, such as loneliness, was associated with the levels of perceived social support among college students. Chinese adolescents desired a fit, self-confident, and self-disciplined characteristic that might be well adapted to the social desirability in the Chinese culture. On the other hand, the western adolescents desired an honest, responsible, and cheerful characteristic [21]. Hong Kong had been a British colony for nearly one hundred years before 1997, and has the longest exposure to the Western culture among the Chinese regions compared with Taiwan, as well as China, which had the least [22]. The differences in quality of life and psychosocial well-being between these Chinese regions have yet to be explored.

With regard to Japanese adolescents, there has been an increasing trend of mental health issues in the past decade [23]. Taijin kyofusho is a culture-specific syndrome which belongs to one type of social anxiety disorder. The symptoms include the anxiety of being offensive to the others in terms of appearance and actions and the avoidance of interpersonal situations [24]. This kind of social withdrawal has become a life ritual which is culturally bound in Japan and more prevalent in adolescents than adults [25]. Interestingly, the symptoms of Taijin kyofusho have been found to be negatively associated with social support [26]. Nevertheless, the evidence of the associations between quality of life and psychosocial well-being among Japanese adolescents was rather limited when compared with neighbor regions.

Concerning adolescents in Southeast Asia, country-specific factors might contribute to the differences in quality of life and psychosocial well-being between countries. A typical example is the Thai who suffered the disaster of the Indian Ocean tsunami in 2004. Thai families suffered physical injuries, psychological trauma and loss of business [27]. These factors were shown to have long-lasting effects on mental health among the Thai population [28,29]. Thai adolescents who were raised in these families required long-term assistance including psychological and financial support in the development of tsunami-resilient communities. Another example is the Filipinos who were subjected to problems in social and school functioning. The Philippines had a relatively high school truancy rate among the Southeast Asian countries [30] and childhood physical violence was prevalent [31]. These country-specific conditions might have negative influences on the quality of life and psychosocial well-being among adolescents.

Improving and maintaining quality of life is considered one of the important goals in promoting health and well-being for adolescents. Quality of life involves several major aspects for ratings such as physical, emotional, and social, as well as school functioning for children and adolescents [32]. In other words, better quality of life would be reflected by better maintenance of physical health and fitness, lifestyle, social support and connection, and control over emotions and conduct in a socioeconomic context. There are several cross-sectional and longitudinal surveys which have studied the associations between quality of life and body weight, sleep outcome, social support and cohabitants, as well as income, self-esteem, lifestyle, and emotional, social and behavioral problems in East Asian adolescents, particularly among Chinese.

Physical activity, sleep duration and probably sleep outcomes have been found to be positively associated with quality of life among adolescents. However, the relationship between weight group and quality of life remains unclear. Xu, et al. [33] conducted a survey of 839 Chinese adolescents aged nine to seventeen in 2013. They found that sleep hours and high physical activity levels were positively associated with health-related quality of life, given that age, gender and parental education were statistically controlled. Yi, et al. [34] conducted a survey of 10,007 Chinese adolescents aged twelve to eighteen between 2016 and 2017. They found that the domains of the quality-of-life scale had significant but small associations with body mass index (BMI) and physical fitness when age was adjusted. However, the adolescents were not categorized into weight groups based on BMI by age locally for the analysis.

Social support from family, friends and teachers was found to have a positive association with quality of life in terms of mental health and emotional functioning, which may be age dependent. On the contrary, social and behavioral problems were negatively associated with quality of life in terms of psychosocial well-being and even the perceived physical appearance, which might be related to self-esteem. Tian, et al. [35] conducted a survey of 361 Chinese adolescents aged twelve to seventeen. They found that a friend’s support was positively associated with well-being at school in terms of positive affect in older adolescents but negative affect in younger ones when age, gender and school grade were statistically adjusted. Teachers’ support had a positive effect on satisfaction at school and on the emotions of students, particularly among younger students. Apart from that, the deterioration in well-being related to schoolwork might result in a lower perceived quality of life. Xu, Chen, Stevens, Zhou, Qi, Wang, Hong, Chen, Yang, Wang and Ratcliffe [33] found that homework which might be associated with academic stress had a negative relationship with quality of life among Chinese adolescents. Wu, et al. [36] conducted a survey of 459 Chinese adolescents aged thirteen to eighteen in 2015. They found that the mediation effect of social support between cohabitants and quality of life in terms of mental health was statistically significant after controlling for gender and ethnicity. Lin, et al. [37] analyzed the data of 5488 Taiwanese adolescents aged eleven to eighteen from the Project for the Health of Children and Adolescents in 2009. They found that peer and conduct problems such as victimization of bullying had negative effects on quality of life in terms of social relationship, perceived physical appearance and psychological well-being, given that age, gender and depressive symptoms were statistically controlled.

Socioeconomic status and family conditions have been found to have significant associations with perceived quality of life among adolescents. Low socioeconomic status was found to be negatively associated with emotional quality of life and self-esteem, but positively associated with risky behaviors among adolescents. On the other hand, whether the child was living with parents or not might have significant impacts on the perceived quality of life in different domains. Shek and Lee [38] analyzed the data of around 2700 Hong Kong adolescents with a mean age of thirteen in a longitudinal study. They found that the poverty level had a significant negative effect on the parent-child relational quality with a small effect size, yet emotional quality of life, including hope, mastery, satisfaction and self-esteem, of adolescents experiencing economic disadvantage was poorer. Six years later, Shek and Lin [39] found that the poverty group by time interaction had a significant effect on substance use with a very small change nonetheless. Huang, et al. [40] conducted a survey of 526 Chinese adolescents aged two to eighteen in 2013. Questionnaires were completed by their caregivers if the children were younger than four years old. They found that parents’ migrant working years had significant negative effects on the child’s health-related quality of life in terms of physical functioning and school performance when age, gender, caregiver, parents’ education level and the number of months of parent visits were statistically controlled. Nakatomi, et al. [41] conducted a survey of 204 Japanese adolescents aged eight to fifteen in 2016. They found that younger students living in homes for children had lower a quality of life in terms of emotional well-being, self-esteem, family and school than students from traditional homes in which parents were living with the children.

Furthermore, recent evidence from a multinational study in East and Southeast Asia showed that lifestyle behaviors related to nutrition, identity awareness and health awareness were significantly associated with quality of life. Lee, Chien, Tanida, Takeuchi, Rutja, Kwok and Lee [19] conducted a survey of 2296 adolescents aged nine to sixteen in China, Hong Kong, Japan, South Korea and Thailand in 2017. They found that physical participation, nutrition, social support, identity awareness and health practices were significantly associated with quality of life, given that country, age, gender, education level, religious belief and parents’ marital status were statistically adjusted. However, the associations between quality of life and weight status, sleep outcomes such as daytime sleepiness, social support by age and living with parents require further investigation, particularly beyond Chinese adolescent populations.

The aim of this survey was to examine the associations between quality of life and body weight, sleep outcome, social support by age and cohabitants given that income, self-esteem, lifestyle, and emotional, social and behavioral problems were taken into consideration among adolescents in East and Southeast Asia. The hypothesis of the current study was that the normal weight status, less daytime sleepiness, more perceived social support by age, and living with parents have significant positive associations with perceived quality of life when income, self-esteem, lifestyle, and emotional, social and behavioral problems are statistically controlled for.

## 2. Methods

### 2.1. Study Design and Settings

This study was a cross-sectional survey conducted in Zhengzhou and Hong Kong SAR of mainland China (PRC), Taipei of Taiwan (ROC), Kansai region of Japan, Bangkok of Thailand and Metro Manila of the Philippines. The settings of the survey were primary schools, secondary schools and homes. The adolescent respondents and their parents were recruited, and data were collected between 2016 and 2017 in their home country.

### 2.2. Participants and Eligibility Criteria

The inclusion criteria involved adolescents who were (1) studying at grade four to grade eleven; (2) living in the city area; and (3) proficient in reading and writing in their own mother language. The researchers from the World Health Organization Collaborating Centers in each city invited schools and recruited respondents locally by convenience sampling method.

### 2.3. Variables

The demographic variables included age in years, height and weight, gender, school grade, religious belief, cohabitants, parents’ marital status and education level, and monthly household income. The psychosocial variables included the health-related quality of life; health-related behaviors such as lifestyle and daytime sleepiness; psychosocial well-being such as emotion and behavioral issues, self-esteem, and perceived social support.

### 2.4. Data Sources

Upon receiving written consent from parents and the implied consent from students, the schoolteachers distributed the demographic questionnaire and the psychometric questionnaire to each adolescent participant. The psychometric scales in the questionnaire were completed independently by the adolescents at school. They also brought the demographic questionnaires for parents to complete at home. The local research team collected the completed questionnaires via the schoolteachers at each participating school. Those who did not participate in the survey undertook other usual activities at school.

### 2.5. Measurement

Age in years, height and weight were assessed in open-ended items. The body mass index was computed by dividing the weight in kilograms by height in meters squared. The body mass indexes of the participants were categorized into (1) overweight or obese, or (2) not overweight at the 85th percentile which was specific for age, gender and country among adolescents in China [42], Hong Kong [43], Taiwan [44] and Japan [45]. The reference from World Health Organization [46] was used for the Filipinos as there was limited related studies. The Thai did not report height and weight.

Gender, education level, religious beliefs, cohabitants, parents’ marital status and education level were assessed on categorial scales. Monthly household income was also assessed on a categorial scale of five quintiles in 2016 which were identified with reference to the government’s studies and reports in China [47], Hong Kong [48], Taiwan [49], Japan [50] and the Philippines [51], as well as the central bank’s study in Thailand [52]. The first two quintiles were considered low-income groups.

The Pediatric Quality of Life Questionnaire 4.0 (QOL) [53] was used to assess the quality of life of the adolescent participants. The twenty-three items were categorized into four subscales including Physical, Emotional, Social, and School Functioning. Each item was scored on a five-point frequency scale (1 = Never, to 5 = Almost always). All items were negatively keyed items. The scores of the items were reversed so that higher score means better quality of life. The Cronbach’s alpha of the subscales in our sample ranged between 0.72 and 0.81. The convergent and factorial validity of the Chinese version [54], the known groups validity of the Japanese version [55] and the construct validity of the Thai version [56] were satisfactory. The intra-rater reliability of the instrument was acceptable among Chinese (ICC = 0.67 to 0.84) [54], Japanese (*r* = 0.46 to 0.73) [55] and Thai (ICC = 0.66 to 0.73) [56] adolescents.

The Multidimensional Scale of Perceived Social Support (PSS) [57] was used to assess perceived social support among the adolescents. There were twelve items which subjectively assessed the perceptions of social support adequacy from three different sources, namely family, friends, and significant others. Each item was scored on a seven-point Likert scale (1 = Very strongly disagree, to 7 = Very strongly agree). All items were positively keyed items. A higher score means better social support. The Cronbach’s alpha of the subscales in our sample ranged between 0.88 and 0.90. The factorial and concurrent validity were acceptable in the Chinese version [58].

The Pediatric Daytime Sleepiness Scale (PDS) [59] was adopted to measure the daytime sleepiness of the adolescents. There were eight items on the instrument. Each item was scored on a five-point scale (1 = Never, to 5 = Always). There were seven items which were negatively keyed. The scores of negatively keyed items were reversed so that a higher score means being less sleepy during the daytime. The Cronbach’s alpha of the scale in our sample was 0.80. The discriminative validity of the Chinese version [60] and the factorial validity and concurrent validity of the Japanese version [61] were acceptable. The correlation coefficient of test-retest reliability of the Chinese version was 0.78 [60] which was satisfactory. The intra-class correlation coefficient of intra-rater reliability of the Japanese version was 0.88 [61].

The Strengths and Difficulties Questionnaire (SDQ) [62] was a brief behavioral screening questionnaire used to assess attributes in relation to emotion and behavioral issues. The twenty-five self-rating items were categorized into five domains of emotions, conduct, attention deficiency/hyperactivity, peer relationships, and prosocial behaviors. Each item was scored on a five-point frequency scale (1 = Not true, to 3 = Certainly true). There were fifteen negatively keyed items. The scores of the negatively keyed items were reversed so that a higher score means better psychosocial well-being. The Cronbach’s alpha of the subscales in our sample ranged between 0.58 and 0.70, except for the peer subscale (0.30). The factorial, convergent and discriminant validity as well as test-retest reliability were acceptable among Chinese adolescents (*r* = 0.71) [63].

The Rosenberg Self-Esteem Scale (RSE) [64] was used to assess adolescents’ internally generated self-esteem and feelings about themselves. There were ten items on the instrument. The participants indicated how strongly they agreed or disagreed with each statement on a four-point scale (1 = Strongly agree, to 4 = Strongly disagree). Half of the items were positively keyed items. The scores of the positively keyed items were reversed so that a higher score means better self-esteem. The Cronbach’s alpha of the scale in our sample was 0.72. The factorial validity among Chinese adolescents was acceptable [65].

The Adolescent Lifestyle Questionnaire (ALQ) [66] was used to measure the health-related lifestyle behaviors of adolescents. The forty-three items were grouped into seven dimensions, namely self-identity, nutrition habits, physical participation, safety, health awareness, social support, and stress management. Items 38 and 40 related to sexual behavior and were withheld. The participants indicated how often they adopt specific healthy lifestyle practices on a five-point frequency scale (1 = Never, to 5 = Almost always). All items were positively keyed items. A higher score means better lifestyle. The Cronbach’s alpha of the subscales in our sample ranged between 0.65 and 0.87. The factor analysis identified the seven dimensions and the intra-rater reliability was satisfactory (*r* = 0.8 to 0.88).

The English version of the psychometric scales for assessing perceived quality of life, social support, daytime sleepiness, strengths and difficulties, self-esteem and lifestyle were translated into the local language of each country by the researchers at the WHO collaborating centers locally, except for the Filipinos who used the English version. In each city, the first bilingual translator translated the English version into the local language, then the second translator back translated the local version into English. The third translator compared both English versions and discussed with the other two translators to revise the local version until all of them agreed that the similarities in the semantic and conceptual meaning between the English version and the local version were the highest in the local cultural context.

### 2.6. Statistical Analysis

IBM SPSS Statistics for Windows, Version 25.0. (IBM Corp., Armonk, NY, USA) was used for statistical analysis. Regarding demographic characteristics, the scale variables such as age and body mass index between cities were compared by using a one-way analysis of variance. The categorical variables such as gender and weight status between cities were compared by using a chi-squared test. Generalized estimating equations were used to estimate parameters of each domain of quality of life in four regression models with different combinations of fixed effects. The fixed effects were gender, monthly household income, age, perceived social support by age, daytime sleepiness, emotion and behavioral control, self-esteem, and lifestyle, with or without the fixed effects of city (country), weight status or living with parents. The probability distribution of the response was assumed as Gaussian, therefore the identity link function was used. The scale parameter was estimated with the maximum-likelihood method. The hypothesis test of the model effects was type III analysis of Wald chi-squared. The sequential Bonferroni method was used to adjust the p values in multiple comparisons of estimated marginal means of the domains of quality of life. The R-squared was computed as one minus the quotient of dividing the scale parameter by the variance of the response in each model [67]. The significance level was set at 0.001 because of the large sample size.

### 2.7. Ethical Considerations

Ethical approval was obtained by each participating institution from the University Ethical Committee or Institution Review Board in each city (country). The principles of the Declaration of Helsinki [68] were strictly followed. Information sheets and consent forms which explained the study purpose, procedure, potential risks and participants’ rights were delivered to parents by the schools. Parental written consents were obtained for all participants under eighteen years old via the schools’ communication system. The adolescents and parents could withdraw at any time without penalty. To ensure confidentiality, respondents were not required to report their names on the questionnaires, and names of the schools and participants would not be disclosed to the third parties. The questionnaires and the electronic data were read and stored by the researchers only.

## 3. Results

### 3.1. Demographic Characteristics

Table 1 shows that there were significant differences in demographic variables between the six cities (countries) (*p* < 0.001). The total number of adolescent respondents was 21,359. The mean age ranged between 12 and 13 years old across cities. The gender ratio was 1:1 except in Kansai region of Japan and Manila of the Philippines in which the percentage of female was higher than male by eight and sixteen per cent, respectively. There were 21 to 29 per cent of adolescent respondents who were categorized as overweight or obese in China, Taiwan and the Philippines. The percentages of those who were not overweight in Kansai region of Japan and Hong Kong were 94 and 88 per cent respectively. A majority of the respondents were living with parents. There were 79 to 84 per cent of adolescents who lived with parents in Kansai region, Hong Kong, Zhengzhou of China and Taipei. The percentage was somehow lower in Bangkok which was 69 per cent. The proportions of those whose parents had separated, divorced or deceased was higher in Thailand, the Philippines and Taiwan and were 32, 27 and 22 per cent, respectively. Nineteen to 39 per cent of the parents received tertiary education across countries. In respect of monthly household income, 72 per cent of the Hong Kong adolescent respondents were living in households with a monthly income lower than the third quintile locally. Conversely, 75 per cent of the Chinese had an income at the third quintile or above. In addition, 53 and 51 per cent of the households generated an income below the third quintile among Thai and the Taiwanese, respectively. Furthermore, 35 and 33 per cent of the Japanese and the Filipino households respectively had an income lower than the third quintile. In addition, the percentages of missing data of age, gender, education level, family monthly income, parents’ marital status, and the domain scores of all the psychometric scales ranged between 0.6 and 7.8 per cent, which were small.

### 3.2. Associations between the Quality of Life and the Demographic and Psychosocial Variables

Table 2 shows the results of four models in which physical functioning of quality of life regressed on different combinations of independent variables. When weight status was added into the models, overweight or obesity was associated with lower physical functioning (B = −0.07, *p* < 0.001) as compared with not being overweight even when country effect was statistically controlled. Less daytime sleepiness was associated with better physical functioning in all models (B = 0.1, *p* < 0.001). Nonetheless, there were no significant results between physical functioning and living with parents or perceived social support by age. Age was significantly associated with physical functioning when the effect of cities was not included. However, the effects were small (B = −0.02, *p* < 0.001). On the other hand, emotion and behavioral control showed stronger positive association with physical functioning (B = 0.54 to 0.57, *p* < 0.001) than lifestyle and self-esteem (B = 0.1, *p* < 0.001) and even other variables. Interestingly, males had a higher perceived physical functioning than females (B = 0.1, *p* < 0.001). Among the six cities, Kansai region of Japan had the highest level of perceived physical functioning, while Hong Kong and Bangkok were the lowest.

Table 3 shows the regression coefficients and estimated marginal means of emotional functioning of quality of life in the four models. Less daytime sleepiness was positively associated with better emotional functioning (B = 0.2, *p* < 0.001). Age, but not perceived social support by age, showed some significant negative associations with emotional functioning, but the effects were small (B = −0.02 to −0.04, *p* < 0.001). There were no significant differences in emotional functioning between groups of weight status or living with parents or not. Among other fixed effects, emotional and behavioral control had a much stronger positive association with emotional functioning (B = 1, *p* < 0.001). Higher self-esteem also had a positive association with better emotional functioning (B = 0.23 to 0.35, *p* < 0.001). Apart from that, males showed better emotional functioning than females (B = 0.13 to 0.18, *p* < 0.001). Moreover, lifestyle showed a significant association with emotional functioning when the effect of cities, weight status and living with parents were not included. However, the effect was small (B = −0.05, *p* < 0.001). The Japanese Kansai region had the highest mean score of perceived emotional functioning, while the score in Manila was the lowest.

Table 4 shows the regression results of social functioning of quality of life. Overweight or obesity, as well as living in a household with a monthly income lower than the third quintile, was associated with poorer social functioning when the effect of cities were not taken into consideration (B = −0.06, *p* < 0.001). Moreover, living with parents was not significantly associated with social functioning. Both age and perceived social support by age had significant but weak associations with social functioning. The associations with age were negative (B = −0.02 to −0.05, *p* < 0.001) but positive with perceived social support by age (B = 0.002 to 0.004, *p* < 0.001). Less daytime sleepiness was positively associated with better social functioning only when the effects of cities (countries), weight status and living with parents were not considered. However, the effect was small (B = 0.04, *p* < 0.001). Aside from that, better emotion and behavioral control had a positive association with social functioning (B = 1, *p* < 0.001) which was stronger than the other effects. Self-esteem was also positively associated with social functioning of quality of life (B = 0.14 to 0.19, *p* < 0.001). However, lifestyle was not significantly associated with social functioning. The Japanese Kansai region had the highest level of perceived social functioning among all cities, while Manila and Bangkok were lower than the other cities.

Table 5 shows the results of regressions of school functioning on demographic, physical and psychosocial variables. Overweight or obesity was associated with worse school functioning as compared with not being overweight when the effect of cities was not statistically controlled (B = −0.06, *p* < 0.001). Living with parents was associated with better school functioning than not living with parents (B = 0.1, *p* < 0.001). Furthermore, feeling less sleepy during the daytime was associated with better school functioning (B = 0.14 to 0.16, *p* < 0.001). Age, but not perceived social support by age, had significant associations with school functioning. Nonetheless, the effects were small (B = −0.03 to −0.06, *p* < 0.001). Other than that, the better the emotional and behavioral control, the better the perceived school functioning (B = 0.7 to 0.8, *p* < 0.001). Better self-esteem and lifestyle also showed positive associations with school functioning, but the effects were much smaller nonetheless (B = 0.1, *p* < 0.001). The Japanese adolescents in Kansai region had the highest mean score of perceived school functioning among all cities. On the other hand, the perceived school functioning in Manila was the lowest.

## 4. Discussion

The aim of this survey was to examine the associations between quality of life and body weight, sleep outcome, social support by age and cohabitants, given that income, self-esteem, lifestyle, and emotional, social and behavioral problems were taken into considerations among adolescents in East and Southeast Asia. The study hypothesis that normal weight status, less daytime sleepiness, and living with parents would have significant positive associations with perceived quality of life was supported by the current results with regard to different domains of quality of life. However, the hypothesis of the association between perceived social support by age and quality of life was not supported.

According to the current results, overweight or obesity was associated with lower physical, social and school functioning with small effects when compared with not being overweight. Yi, Fu, Burns and Ding [34] previously found that the domains of quality of life scale had significant but small associations with body mass index among Chinese adolescents which were grouped according to international cutoffs. However, the reference of the cutoffs was unclear. Instead, the body mass index should be categorized with reference to age, gender and ethnic group or country. The results of the current study show that those who are overweight or obese may have worse perceived quality of life than those who are not overweight among adolescents.

Besides, the current results show that less daytime sleepiness was found to be associated with better physical, emotional and school functioning among adolescents in East and Southeast Asia. Xu, Chen, Stevens, Zhou, Qi, Wang, Hong, Chen, Yang, Wang and Ratcliffe [33] found that sleep hours had a positive association with quality of life assessed in nine dimensions including Daily Routine, Ability to Join in Activities, Sleep, Worried, Sad, Pain, Tired, Annoyed and Schoolwork among Chinese adolescents. The current results are consistent with the previous study in that sleep may have significant influence on perceived quality of life in terms of physical, emotional and school functioning among adolescents.

Nevertheless, the significant associations between lifestyle and physical and school functioning became weaker when other stronger variables such as emotion, behavioral control and self-esteem were statistically adjusted in the current study. Earlier, Lee, Chien, Tanida, Takeuchi, Rutja, Kwok and Lee [19] found that the lifestyle domains of physical participation, nutrition and health awareness had weaker associations with the overall quality of life than other domains such as social support and identity awareness. However, perceived social support by age only shows minimal association with social functioning of quality of life when age and other variables were statistically controlled in the current study. Tian, Liu, Huang and Huebner [35] found that a friend’s support was positively associated with negative affect in younger adolescents but positive affect in older adolescents among Chinese. The results of the current study and the literature imply that the rating of social support received might not show strong association with either better or worse perceived quality of life if it links to both positive and negative experiences and emotions.

On the other hand, the notable results were that the associations between emotional and behavioral control and quality of life were the strongest in all statistical models in the current study. Lin, Yen, Lin, Wang, Liu, Hu and Yen [37] found that peer and behavioral problems such as victimization of bullying had negative effects on quality of life in terms of social relationship, perceived physical appearance and psychological well-being among Taiwanese adolescents. The poorer perceived physical appearance might also be associated with lower self-esteem [69,70]. The results of the current study show that better self-esteem consistently had significant positive associations with all domains of quality of life. Hence, when the self-rating of emotion, behaviors and self-esteem become better, the level of perceived quality of life could be higher among East and Southeast Asian adolescents.

Several demographic characteristics show significant associations with quality of life in the current study which found that living with parents was associated with better school functioning than not living with parents. Huang, Zhong, Li, Xu, Zhang, Feng, Yang, Bo and Deng [40] found that parents’ migrant working years had significant negative effects on the child’s physical functioning and school performance among Chinese adolescents when parents’ visit intervals were statistically controlled. Nakatomi, Ichikawa, Wakabayashi and Takemura [41] found that younger Japanese adolescents living in homes for children had lower quality of life in terms of emotional well-being, self-esteem, family and school than those who were living with parents without adjusting for other variables in regression models. Notwithstanding, living with parents is likely an important contributing factor to better school functioning of quality of life among East Asian adolescents.

Apart from that, having a monthly household income below the third quintile was associated with poorer social functioning when the effect of cities was not controlled in the current study. Shek and Lee [38] found that Hong Kong adolescents who experienced economic disadvantage had worse quality of life in terms of emotional well-being and self-esteem without adjusting for other variables. After all, the effect of income groups or poverty levels on perceived quality of life among adolescents was very small.

Concerning the gender difference in quality of life, the current study found that males had better physical and emotional functioning than females after adjusting for other significant variables among East and Southeast Asian adolescents. Huang, Zhong, Li, Xu, Zhang, Feng, Yang, Bo and Deng [40] found that the left-behind males had lower school functioning than females in rural China when the parents’ migrant working years and the number of months of parent visits were statistically controlled. The different findings between studies may be related to the differences in the target populations, countries considered, the time of conducting the study, and the variables included in the statistical models. Based on the results of the current study in six cities (countries), there were no significant differences in social and school functioning between genders. In respect to the difference in quality of life between cities, Kansai region of Japan had the highest perceived quality of life in all domains. On the contrary, Manila was the lowest in emotional, social and school functioning, while Bangkok was among the lowest in physical and social functioning.

## 5. Limitations

The self-administered questionnaire was subjected to social desirability bias and recall bias. Moreover, there may be selection bias as the participants were conveniently recruited. Furthermore, time-varying effects were not considered in this cross-sectional study. Thus, there may be limitations on the generalizability of the study results to the entire country within China, Japan, Thailand and the Philippines, respectively.

## 6. Interpretation

Adolescents who are overweight or obese are more likely to experience poorer physical, social and school functioning of quality of life than those who are not. Those who have better sleep outcomes performed better in physical, emotional and school functioning. In addition, better lifestyle was significantly associated with better physical and school functioning. Weight management and sleep hygiene are considered as crucial in maintaining quality of life among East and Southeast Asian adolescents. More importantly, adolescents who have better self-esteem and control of emotions and behaviors have much a higher level of perceived quality of life in all domains. On the other hand, living with parents was found to have significant associations with quality of life in the domains of emotional functioning, family functioning and particularly school functioning. Family and parenting may play a significant role in a child’s health, education and socialization [71].

## 7. Implications

Further studies are required to explore the differences in perceived quality of life between genders and countries. First, the results from one study, as well as a cross-sectional study, may not be conclusive. The second issue is that there is limited evidence of measurement invariance between genders and countries in Asia except for a study by Lin, Luh, Yang, Su, Wang and Ma [54] that found that the gender invariance of the Chinese version of the Pediatric Quality of Life Inventory 4.0 was acceptable. Furthermore, the conceptual framework revised based on the current results may be applied to other countries globally to examine its generalizability and external validity. The evidence in the current study could be used to develop regional goals and action plans to promote health-related quality of life for East and Southeast Asian adolescents. Interventional studies and implementation research of the related aims should be further reviewed and revised accordingly.

In addition, future studies are needed for a better understanding of the predictors of health risks and protective behaviors with both positive and negative health outcomes for adolescents in the Asian regions. Moreover, the differences in health status between different patterns of socio-demographic characteristics suggest future research and policy making in promoting psychosocial health among adolescents in Asia Pacific region [18]. A follow-up survey will be conducted between September 2020 and 2021 in the six regions of this study to yield new longitudinal evidence.

## 8. Significance

This is the first study to estimate and compare the parameters of quality of life, psychosocial well-being and health-related behaviors of adolescents in six Asia Pacific regions including China, Hong Kong, Taiwan, Japan, the Philippines, and Thailand. The adolescent health and behavior patterns sustain a rather comprehensive conceptual picture of adolescence. The utilization of the evidence should be aimed at facilitating policy makers, health planners, health educators and health promoters to (1) strategize and incorporate up-to-date research findings, (2) plan and implement relevant programs and services, (3) provide early interventions, (4) meet health care needs, and (5) reduce morbidity and premature death rates of adolescents in specific countries. The use of the evidence may also facilitate the achievement of the United Nation’s Sustainable Development Goal in promoting health and well-being and reducing diseases among adolescents, as well as the analysis of cost-effectiveness of interventions to prioritize them with evidence-based strategies.

## 9. Conclusions

In conclusion, psychosocial well-being is shown to be positively associated with quality of life when region and demographic factors are statistically controlled. Hence, better psychosocial well-being might be a priority in the promotion of quality of life among adolescents in the Asia-Pacific region. Nevertheless, further studies are required to explore significant contributing factors to better quality of life among Asian adolescents.

## Figures and Tables

**Table 1:** Demographic characteristics of the adolescent participants and their families.

Characteristics	Category	Philippines *N* = 4536	Thailand *N* = 3156	China *N* = 6401	Hong Kong *N* = 3727	Taiwan *N* = 1611	Japan *N* = 1928	*F*(*df*)	*p*
		Mean (SD)	Mean (SD)	Mean (SD)	Mean (SD)	Mean (SD)	Mean (SD)		
Age in years		13.3 (1.6)	12.7 (1.7)	12 (1.7)	13.1 (1.9)	12.9 (1.8)	12.2 (1.7)	387.6 (5)	<0.001
Body mass index		18.9 (4.3)	-	19.3 (4.1)	18.6 (3.3)	19.7 (4.1)	17.8 (2.6)	75.9 (4)	<0.001
		***N*** **(%)**	***N*** **(%)**	***N*** **(%)**	***N*** **(%)**	***N*** **(%)**	***N*** **(%)**	**χ^2^**	***p***
Gender	Male	1905 (42)	1561 (49.5)	3390 (53)	1770 (48.8)	787 (49.3)	848 (46.1)	135.1	<0.001
	Female	2631 (58)	1591 (50.5)	3004 (47)	1860 (51.2)	810 (50.7)	991 (53.9)		
Education	Primary school	998 (22)	1453 (46.2)	3601 (56.3)	1392 (37.6)	561 (35.8)	956 (51.7)	1428.4	<0.001
	Secondary school	3538 (78)	1694 (53.8)	2794 (43.7)	2313 (62.4)	1008 (64.2)	892 (48.3)		
Religion	No religion	39 (0.9)	0 (0)	5439 (85.8)	2586 (70.1)	806 (55.2)	-	28,896.5	<0.001
	Protestantism	703 (15.6)	40 (1.3)	298 (4.7)	749 (20.3)	124 (8.5)	-		
	Catholicism	3536 (78.3)	10 (0.3)	42 (0.7)	174 (4.7)	37 (2.5)	-		
	Buddhism	10 (0.2)	3042 (96.8)	444 (7)	128 (3.5)	330 (22.6)	-		
	Islam	32 (0.7)	50 (1.6)	84 (1.3)	17 (0.5)	3 (0.2)	-		
	Other religion	198 (4.4)	1 (0.03)	32 (0.5)	33 (0.9)	161 (11)	-		
Weight status	Overweight or obese	476 (21.1)	-	1711 (28.8)	395 (12.3)	349 (22.9)	90 (5.7)	593.4	<0.001
	Not overweight	1784 (78.9)	-	4225 (71.2)	2817 (87.7)	1175 (77.1)	1482 (94.3)		
Live with grandparents	Yes	-	860 (27.3)	848 (13.3)	578 (15.5)	499 (31)	179 (9.3)	588.5	<0.001
	No	-	2291 (72.7)	5549 (86.7)	3149 (84.5)	1112 (69)	1749 (90.7)		
Live with parents	Yes	-	2166 (68.6)	5259 (82.4)	3108 (83.4)	1270 (78.8)	1540 (84.3)	326.6	<0.001
	No	-	990 (31.4)	1123 (17.6)	618 (16.6)	341 (21.2)	286 (15.7)		
Parents’ marital status	Married	3166 (73.4)	2117 (68.3)	5779 (90.5)	2694 (83.8)	1184 (77.7)	1518 (85.1)	1558.5	<0.001
	Separated	593 (13.7)	447 (14.4)	266 (4.2)	140 (4.4)	76 (5)	96 (5.4)		
	Divorced	197 (4.6)	406 (13.1)	284 (4.4)	299 (9.3)	227 (14.9)	141 (7.9)		
	Deceased	359 (8.3)	129 (4.2)	55 (0.9)	80 (2.5)	36 (2.4)	28 (1.6)		
Father’s education level	Primary school	417 (9.8)	600 (19.7)	408 (6.4)	297 (8.5)	32 (2.3)	-	1214.1	<0.001
	Junior high school	928 (21.8)	631 (20.7)	1921 (30.1)	852 (24.3)	190 (13.5)	-		
	Senior high school	1241 (29.2)	699 (22.9)	2006 (31.5)	1580 (45.1)	655 (46.6)	-		
	Tertiary education	1664 (39.2)	1123 (36.8)	2041 (32)	771 (22)	529 (37.6)	-		
Mother’s education level	Primary school	306 (7)	653 (21.3)	729 (11.5)	336 (9.6)	44 (3.2)	-	1454.0	<0.001
	Junior high school	1001 (23)	539 (17.6)	1884 (29.7)	807 (23.2)	162 (11.6)	-		
	Senior high school	1338 (30.7)	697 (22.8)	1972 (31.1)	1683 (48.3)	659 (47.4)	-		
	Tertiary education	1714 (39.3)	1170 (38.2)	1753 (27.7)	657 (18.9)	526 (37.8)	-		
Monthly household income	First quintile	254 (6.3)	1282 (40.6)	617 (9.8)	1199 (37.7)	221 (16.3)	354 (21.2)	5589.2	<0.001
	Second quintile	1073 (26.4)	405 (12.8)	962 (15.4)	1090 (34.3)	467 (34.4)	236 (14.2)		
	Third quintile	1184 (29.2)	488 (15.5)	2375 (37.9)	658 (20.7)	468 (34.5)	117 (7)		
	Fourth quintile	1425 (35.1)	538 (17)	2040 (32.6)	161 (5.1)	181 (13.3)	959 (57.6)		
	Fifth quintile	125 (3.1)	443 (14)	273 (4.4)	70 (2.2)	21 (1.5)	0 (0)		

**Table 2:** Regression coefficients and estimated marginal means of physical functioning of quality of life.

Study Variables	B [95% CI]	B [95% CI]	B [95% CI]	B [95% CI]
(Intercept)	2.1 [1.9, 2.3] *	2 [1.9, 2.2] *	1.6 [1.5, 1.8] *	1.8 [1.7, 2] *
Zhengzhou, China	−0.29 [−0.33, −0.25] *	-	0.14 [0.1, 0.17] *	-
Hong Kong	−0.39 [−0.44, −0.35] *	-	0.04 [0.0036, 0.077]	-
Taipei, Taiwan	−0.26 [−0.31, −0.21] *	-	0.17 [0.12, 0.21] *	-
Kansai region, Japan	0	-	0.43 [0.39, 0.47] *	-
Manila, the Philippines	-	-	0.14 [0.1, 0.18] *	-
Bangkok, Thailand	-	-	0	-
Overweight or obese	−0.068 [−0.098, −0.038] *	−0.091 [−0.12, −0.061] *	-	-
Not overweight	0	0	-	-
Live with parents	0.037 [0.0025, 0.071]	0.043 [0.0093, 0.078]	-	-
Not living with parents	0	0	-	-
Male	0.11 [0.088, 0.14] *	0.12 [0.093, 0.14] *	0.11 [0.092, 0.13] *	0.11 [0.09, 0.13] *
Female	0	0	0	0
Income below 3rd quintile	0.0041 [−0.022, 0.03]	−0.031 [−0.055, −0.0066]	0.018 [−0.003, 0.038]	−0.016 [−0.036, 0.0037]
Income at 3rd quintile or above	0	0	0	0
Age	−0.0087 [−0.018, 0.00023]	−0.024 [−0.033, −0.016] *	−0.0081 [−0.015, −0.00089]	−0.015 [−0.023, −0.0084] *
Perceived social support by age	−0.00018 [−0.0012, 0.00083]	0.0012 [0.00024, 0.0022]	−0.00015 [−0.00094, 0.00064]	0.00044 [−0.00034, 0.0012]
Daytime sleepiness	0.11 [0.096, 0.13] *	0.1 [0.083, 0.12] *	0.099 [0.084, 0.11] *	0.1 [0.085, 0.11] *
Emotion and behavior	0.57 [0.49, 0.64] *	0.54 [0.47, 0.61] *	0.57 [0.52, 0.63] *	0.56 [0.51, 0.62] *
Self-esteem	0.085 [0.05, 0.12] *	0.084 [0.049, 0.12] *	0.1 [0.075, 0.13] *	0.1 [0.077, 0.13] *
Lifestyle	0.13 [0.1, 0.16] *	0.13 [0.1, 0.15] *	0.14 [0.11, 0.16] *	0.14 [0.12, 0.16] *
(Scale)	0.27	0.29	0.3	0.31
R^2^	0.35	0.32	0.28	0.25
	**Marginal mean [95% CI]**		**Marginal mean [95% CI]**	
Zhengzhou, China	4.19 [4.17, 4.21]		4.16 [4.14, 4.18]	
Hong Kong	4.08 [4.05, 4.11]		4.06 [4.04, 4.08]	
Taipei, Taiwan	4.22 [4.18, 4.26]		4.19 [4.15, 4.23]	
Kansai region, Japan	4.48 [4.44, 4.52]		4.45 [4.42, 4.48]	
Manila, the Philippines	-		4.16 [4.14, 4.18]	
Bangkok, Thailand	-		4.02 [3.99, 4.05]	

* *p* < 0.001. Score range: Quality of life (1–5); Daytime sleepiness (1–5); Perceived social support (1–7) by age (9–16); Emotion and behavior (1–3); Self-esteem (1–4); Lifestyle (1–5).

**Table 3:** Regression coefficients and estimated marginal means of emotional functioning of quality of life.

Study Variables	B [95% CI]	B [95% CI]	B [95% CI]	B [95% CI]
(Intercept)	0.39 [0.17, 0.62] *	0.32 [0.097, 0.55]	0.35 [0.17, 0.52] *	0.33 [0.16, 0.5] *
Zhengzhou, China	−0.27 [−0.32, −0.22] *	-	−0.094 [−0.13, −0.056] *	-
Hong Kong	−0.29 [−0.34, −0.24] *	-	−0.13 [−0.17, −0.089] *	-
Taipei, Taiwan	−0.075 [−0.14, −0.011]	-	0.082 [0.029, 0.14]	-
Kansai region, Japan	0	-	0.15 [0.1, 0.2] *	-
Manila, the Philippines	-	-	−0.28 [−0.32, −0.24] *	-
Bangkok, Thailand	-	-	0	-
Overweight or obese	−0.0026 [−0.041, 0.036]	−0.025 [−0.063, 0.013]	-	-
Not overweight	0	0	-	-
Live with parents	0.057 [0.015, 0.1]	0.054 [0.011, 0.096]	-	-
Not living with parents	0	0	-	-
Male	0.13 [0.1, 0.16] *	0.14 [0.11, 0.17] *	0.17 [0.14, 0.19] *	0.18 [0.15, 0.2] *
Female	0	0	0	0
Income below 3rd quintile	−0.018 [-0.051, 0.015]	−0.026 [−0.057, 0.0045]	−0.017 [−0.041, 0.0072]	−0.0058 [−0.029, 0.017]
Income at 3rd quintile or above	0	0	0	0
Age	−0.02 [−0.032, −0.0089] *	−0.026 [−0.037, −0.015] *	−0.024 [−0.033, −0.015] *	−0.036 [−0.045, −0.028] *
Perceived social support by age	−0.0011 [−0.0024, 0.0002]	0.00003 [−0.0012, 0.0013]	−0.00078 [−0.0017, 0.00018]	0.00007 [−0.00088, 0.001]
Daytime sleepiness	0.2 [0.17, 0.22] *	0.19 [0.17, 0.21] *	0.17 [0.15, 0.18] *	0.17 [0.15, 0.18] *
Emotion and behavior	0.94 [0.85, 1] *	0.92 [0.82, 1] *	1.1 [0.99, 1.1] *	1.1 [1.1, 1.2] *
Self-esteem	0.35 [0.31, 0.4] *	0.34 [0.3, 0.39] *	0.26 [0.23, 0.29] *	0.23 [0.2, 0.26] *
Lifestyle	−0.033 [−0.068, 0.0026]	−0.04 [−0.075, −0.0053]	−0.019 [−0.046, 0.0079]	−0.054 [−0.08, −0.027] *
(Scale)	0.44	0.45	0.43	0.45
R^2^	0.33	0.31	0.35	0.32
	**Marginal mean [95% CI]**		**Marginal mean [95% CI]**	
Zhengzhou, China	3.75 [3.72, 3.78]		3.68 [3.66, 3.71]	
Hong Kong	3.73 [3.69, 3.76]		3.65 [3.62, 3.68]	
Taipei, Taiwan	3.94 [3.89, 3.99]		3.86 [3.82, 3.91]	
Kansai region, Japan	4.02 [3.97, 4.07]		3.93 [3.89, 3.97]	
Manila, the Philippines	-		3.5 [3.47, 3.52]	
Bangkok, Thailand	-		3.78 [3.75, 3.81]	

* *p* < 0.001. Score range: Quality of life (1–5); Daytime sleepiness (1–5); Perceived social support (1–7) by age (9–16); Emotion and behavior (1–3); Self-esteem (1–4); Lifestyle (1–5).

**Table 4:** Regression coefficients and estimated marginal means of social functioning of quality of life.

Study Variables	B [95% CI]	B [95% CI]	B [95% CI]	B [95% CI]
(Intercept)	2 [−1240.8, 1244.8]	2 [1.8, 2.2] *	1.2 [1.1, 1.4] *	1.3 [1.1, 1.4] *
Zhengzhou, China	−0.2 [−982.7, 982.3]	-	0.24 [0.21, 0.28] *	-
Hong Kong	−0.34 [−1030.8, 1030.1]	-	0.1 [0.067, 0.14] *	-
Taipei, Taiwan	−0.18 [−1162.7, 1162.4]	-	0.25 [0.2, 0.3] *	-
Kansai region, Japan	0	-	0.47 [0.42, 0.51] *	-
Manila, the Philippines	-	-	−0.11 [−0.14, −0.068] *	-
Bangkok, Thailand	-	-	0	-
Overweight or obese	−0.049 [−0.08, −0.017]	−0.058 [−0.089, −0.027] *	-	-
Not overweight	0	0	-	-
Live with parents	0.017 [−0.018, 0.053]	0.022 [−0.014, 0.057]	-	-
Not living with parents	0	0	-	-
Male	−0.0012 [−0.025, 0.023]	0.0029 [−0.022, 0.027]	−0.0077 [−0.028, 0.012]	0.0052 [−0.015, 0.026]
Female	0	0	0	0
Income below 3rd quintile	−0.018 [−0.045, 0.009]	−0.06 [−0.085, −0.035] *	−0.0045 [−0.026, 0.017]	−0.029 [−0.05, −0.0082]
Income at 3rd quintile or above	0	0	0	0
Age	−0.04 [−0.05, −0.03] *	−0.053 [−0.063, −0.044] *	−0.023 [−0.031, −0.015] *	−0.039 [−0.047, −0.031] *
Perceived social support by age	0.0032 [0.0021, 0.0043] *	0.0043 [0.0032, 0.0054] *	0.0023 [0.0015, 0.0032] *	0.0027 [0.0018, 0.0035] *
Daytime sleepiness	0.0043 [−0.015, 0.023]	−0.0037 [−0.023, 0.015]	0.016 [−0.00009, 0.031]	0.036 [0.02, 0.051] *
Emotion and behavior	0.94 [0.86, 1] *	0.92 [0.84, 1] *	1 [0.95, 1.1] *	1.2 [1.1, 1.2] *
Self-esteem	0.18 [0.14, 0.22] *	0.19 [0.15, 0.22] *	0.16 [0.13, 0.19] *	0.14 [0.11, 0.16] *
Lifestyle	0.02 [−0.0087, 0.048]	0.021 [−0.0071, 0.05]	0.037 [0.013, 0.06]	0.013 [−0.0099, 0.037]
(Scale)	0.29	0.3	0.33	0.36
R^2^	0.44	0.42	0.35	0.30
	**Marginal mean [95% CI]**		**Marginal mean [95% CI]**	
Zhengzhou, China	4.42 [−756.63, 765.48]		4.38 [4.36, 4.4]	
Hong Kong	4.29 [−690.46, 699.04]		4.24 [4.21, 4.26]	
Taipei, Taiwan	4.45 [−434.95, 443.84]		4.38 [4.35, 4.42]	
Kansai region, Japan	4.63 [4.63, 4.63]		4.6 [4.57, 4.63]	
Manila, the Philippines	-		4.03 [4, 4.05]	
Bangkok, Thailand	-		4.13 [4.11, 4.16]	

* p < 0.001. Score range: Quality of life (1–5); Daytime sleepiness (1–5); Perceived social support (1–7) by age (9–16); Emotion and behavior (1–3); Self-esteem (1–4); Lifestyle (1–5).

**Table 5:** Regression coefficients and estimated marginal means of school functioning of quality of life.

Study Variables	B [95% CI]	B [95% CI]	B [95% CI]	B [95% CI]
(Intercept)	2 [1.8, 2.2] *	2 [1.8, 2.2] *	1.4 [1.3, 1.6] *	1.6 [1.5, 1.8] *
Zhengzhou, China	−0.45 [−0.49, −0.41] *	-	0.019 [−0.013, 0.051]	-
Hong Kong	−0.5 [−0.54, −0.46] *	-	−0.04 [−0.073, −0.0062]	-
Taipei, Taiwan	−0.47 [−0.52, −0.42] *	-	−0.0054 [−0.05, 0.04]	-
Kansai region, Japan	0	-	0.47 [0.43, 0.51] *	-
Manila, the Philippines	-	-	−0.16 [−0.2, −0.13] *	-
Bangkok, Thailand	-	-	0	-
Overweight or obese	−0.0095 [−0.04, 0.021]	−0.059 [−0.089, −0.029] *	-	-
Not overweight	0	0	-	-
Live with parents	0.082 [0.049, 0.12] *	0.096 [0.062, 0.13] *	-	-
Not living with parents	0	0	-	-
Male	−0.0037 [−0.027, 0.019]	0.0038 [−0.02, 0.028]	−0.022 [−0.041, −0.0036]	−0.014 [−0.033, 0.0055]
Female	0	0	0	0
Income below 3rd quintile	0.016 [−0.0093, 0.042]	−0.0073 [−0.032, 0.017]	0.017 [−0.0034, 0.037]	0.0095 [−0.01, 0.029]
Income at 3rd quintile or above	0	0	0	0
Age	−0.035 [−0.044, −0.026] *	−0.057 [−0.065, −0.048] *	−0.026 [−0.033, −0.019] *	−0.045 [−0.052, −0.038] *
Perceived social support by age	−0.00098 [−0.002, 0]	0.001 [0.00005, 0.002]	−0.0012 [−0.002, −0.00042]	0.00003 [−0.00074, 0.00081]
Daytime sleepiness	0.16 [0.14, 0.18] *	0.14 [0.12, 0.16] *	0.16 [0.14, 0.17] *	0.15 [0.14, 0.17] *
Emotion and behavior	0.72 [0.65, 0.79] *	0.68 [0.6, 0.75] *	0.76 [0.71, 0.82] *	0.84 [0.79, 0.89] *
Self-esteem	0.12 [0.086, 0.16] *	0.11 [0.078, 0.15] *	0.11 [0.087, 0.14] *	0.087 [0.061, 0.11] *
Lifestyle	0.095 [0.066, 0.12] *	0.081 [0.052, 0.11] *	0.093 [0.069, 0.12] *	0.06 [0.037, 0.083] *
(Scale)	0.26	0.29	0.29	0.31
R^2^	0.43	0.38	0.37	0.32
	**Marginal mean [95% CI]**		**Marginal mean [95% CI]**	
Zhengzhou, China	4.05 [4.03, 4.08]		4 [3.99, 4.02]	
Hong Kong	4 [3.97, 4.03]		3.95 [3.92, 3.97]	
Taipei, Taiwan	4.04 [4, 4.07]		3.98 [3.94, 4.02]	
Kansai region, Japan	4.5 [4.47, 4.54]		4.45 [4.42, 4.48]	
Manila, the Philippines	-		3.82 [3.8, 3.85]	
Bangkok, Thailand	-		3.99 [3.96, 4.01]	

* *p* < 0.001. Score range: Quality of life (1–5); Daytime sleepiness (1–5); Perceived social support (1–7) by age (9–16); Emotion and behavior (1–3); Self-esteem (1–4); Lifestyle (1–5).

## Protocol

The reporting follows the STROBE statement [72] which could be assessed at https://www.strobe-statement.org/index.php?id=available-checklists.
